# Supplementary material for: Modulation of calcium signaling and metabolic pathways in endothelial cells with magnetic fields
Source: Nanoscale Adv. 2024 Jan 23;6(4):1163–82. doi: 10.1039/d3na01065a (PMC10863714; doi:10.1039/d3na01065a)
Supplement: NA-006-D3NA01065A-s001 [file NA-006-D3NA01065A-s001.pdf]

Electronic Supplementary Material (ESI) for Nanoscale Advances.
This journal is © The Royal Society of Chemistry 2024

Supplementary material

Magnetic shear stress in the modified Plank's model Ca^{2+} dynamics in endothelial cells taking on account magnetic field influence on the chains of magnetic nanoparticles embedded in cell membrane.

The transformation from dimensional dynamic variables to dimensionless ones is given in Table S1.

Table S1. Dynamic variables in the Plank's model for calcium dynamics.

Dynamic variable	Dimensional notation	Dimensionless notation
Concentrations of IP3 in the cytosol	$\mathbb{i}$	$i^{'} = \frac{\mathbb{i}}{K_4}$
Free Ca^{2+} in the cytosol	Ca_c, μM	$Ca_c^{'} = \frac{Ca_c}{K_4}$
Buffered Ca^{2+} in the cytosol	Ca_b, μM	$Ca_b^{'} = \frac{Ca_b}{K_4}$
Ca^{2+} in the internal stores	Ca_s, μM	$Ca_s^{'} = \frac{Ca_s}{K_4}$
Sum of free Ca^{2+} in the cytosol and buffered Ca^{2+} in the cytosol	$Ca_t = Ca_c + Ca_b$, μM	$Ca_t^{'} = \frac{Ca_t}{K_4}$

Let's take on account

$$2Ca_c = Ca_t - B_T - \frac{k_7}{k_6} + \left(\left(Ca_t - B_T - \frac{k_7}{k_6}\right)^2 + 4\frac{k_7}{k_6}Ca_t\right)^{1/2} \qquad \text{(S1)}$$

Substituting (S1) into the set of equations (10)-(13) we obtain:

$$\frac{di}{dt} = k_1 \frac{\phi}{K_c + \phi} \cdot \frac{Ca_c}{K_1 + Ca_c} - k_2 i \qquad \text{(S2)}$$

$$\frac{dCa_t}{dt} = k_3 \frac{Ca_c}{K_{CICR} + Ca_c} \left(\frac{i}{K_2 + i} \right)^3 Ca_s - k_4 \left(\frac{Ca_C}{K_3 + Ca_c} \right)^2 + k_5 Ca_s^2 + k_{CCE} \left(Ca_{s,0} - Ca_s \right) \left(Ca_{ex} - Ca_c \right) + \frac{\text{[illegible]}}{1 + \alpha \text{[illegible]}} - \frac{k_8}{K_4 + Ca_c} \qquad \text{(S3)}$$

$$\frac{dCa_s}{dt} = -V_r \left(k_3 \frac{Ca_c}{K_{CICR} + Ca_c} \left(\frac{i}{K_2 + i} \right)^3 Ca_s - k_4 \left(\frac{Ca_C}{K_3 + Ca_c} \right)^2 + k_5 Ca_s^2 \right) \qquad \text{(S4)}$$

The initial conditions are used for the equations (12)-(14):

$$i(0) = 0 \qquad \text{(S5)}$$

$$Ca_t(0) = Ca_0 \left(\frac{Ca_0 + B_T + k_7 / k_6}{Ca_0 + k_7 / k_6} \right) \qquad \text{(S6)}$$

$$Ca_s(0) = Ca_{s,0} \qquad \text{(S7)}$$

The non-dimensionless equations have the form after non-dimensionalisation (Table S1, Table S2):

$$\frac{di'}{dt'} = k_1' \frac{\phi'}{K_c' + \phi'} \cdot \frac{Ca_c'}{K_1' + Ca_c'} - k_2' i' \qquad \text{(S8)}$$

$$\frac{dCa_t'}{dt'} = k_3' \frac{Ca_c'}{K_{CICR}' + Ca_c'} \left(\frac{i'}{K_2' + i'} \right)^3 Ca_s' - k_4' \left(\frac{Ca_c'}{K_3' + Ca_c'} \right)^2 + k_5' Ca_s'^2 + k_{CCE}' \left(Ca_{s,0}' - Ca_s' \right) \left(Ca_{ex}' - Ca_c' \right) + \frac{\text{[illegible]}}{1 + \alpha \text{[illegible]}} - \frac{k_8'}{K_4' + Ca_c'} \qquad \text{(S9)}$$

$$\frac{dCa_s^{'}}{dt^{'}} = -V_r^{'}\left(k_3^{'}\frac{Ca_c^{'}}{K_{CICR}^{'} + Ca_c^{'}}\left(\frac{i^{'}}{K_2 + i^{'}}\right)^3 Ca_s^{'} - k_4^{'}\left(\frac{Ca_c^{'}}{K_3^{'} + Ca_c^{'}}\right)^2 + k_5^{'}Ca_s^{'2}\right) \qquad \text{(S10)}$$

$$W^{'}(\tau^{'}) = \frac{f_e W_0^{'}\left[\varepsilon^{'}\tau^{'} + \sqrt{16\delta^{'2} + \varepsilon^{'2}\tau^{'2}} - 4\delta^{'}\right]^2}{\left[\varepsilon^{'}\tau^{'} + \sqrt{16\delta^{'2} + \varepsilon^{'2}\tau^{'2}}\right]}. \qquad \text{(S11)}$$

The results of numeric solving of equations (S8)-(S11) using Python programming language, numpy and scipy packages.

Table S2. Parameters in the Plank's model for calcium dynamics modified to take on account the WSS induced by magnetic field.

Parameter used in the model for calcium dynamics	Dimensional parameter values for non-oscillatory regime	Relation between dimensional and dimension-less parameter	Dimension-less parameter values for non-oscillatory regime	Dimension-less parameter values for oscillatory regime
Maximum value of spatial derivative of magnetic field flux density	$G_0 = 30\ Tm^{-1}$			
Magnetic field flux density of	$B = 0.5 \cdot 10^{-4}\ T$			

the Earth				
Biogenic or nonbiogenic magnetic nanoparticle radius	$r = 100 \cdot 10^{-9} m$			
Magnetization of biogenic or nonbiogenic magnetic nanoparticle	$M_s = 510 \cdot 10^3 Am^{-1}$			
Frequency of external gradient magnetic field oscillation	$\omega = 4 \cdot 10^{-4} s^{-1}$	$\omega' = \frac{\omega}{T}$	$\omega' = 0.2$	
Amplitude of magnetic WSS (WSS) induced by gradient magnetic field	$P_0 = 2.04\ Pa$ $P_0 = \frac{4rM_s G_0}{3}$	$\tau'_{magn} = \frac{P_0}{\rho U_0^2}$	$\tau'_{magn} =$ 18.9 for artery (Plank's model) $\tau'_{magn} = 906.7$ for capillary	
Amplitude of magnetic WSS	$P_0 = 1.3\ Pa$ $P_0 = \frac{\pi}{6N} M_S B \sin\theta sin\gamma$	$\tau'_{magn} = \frac{P_0}{\rho U_0^2}$	$\tau'_{magn} =$ 12.3 for artery	

(WSS) induced by uniform magnetic field			(Plank's model) $\tau_{magn}^{'} = 593$ for capillary	
Angle between the normal to the cell membrane and magnetic field	θ			
Angle between in-plane component of magnetic field and magnetization of the chain of magnetic nanoparticles	γ			
Concentration of ATP at the cell surface	ϕ [1]	$\phi^{'} = \frac{\phi}{\phi_0}$	$\phi^{'} = 0.9$ [1]	
Reference ATP concentration	$\phi_0 = 0.1\ \mu M$ [1]			
IP3 production		$k_1^{'} = \frac{k_1 T}{K_4}$	$k_1^{'} = 8.53$ [1]	$k_1^{'} = 35.8$ [1]

rate	$k_1 = 5.46 \cdot 10^{-3}\,\mu M s^{-1}$ [1]			
IP3 decay rate	$k_2 = 0.2\,s^{-1}$ [1]	$k_2^{'} = k_2 T$	$k_2^{'} =$ 100 [1]	
Ca^{2+} release rate	$k_3 = 6.64\,s^{-1}$ [1]	$k_3^{'} = k_3 T$	$k_3^{'} = 3320$ [1]	
Ca^{2+} resequestration rate	$k_4 = 5\,\mu M s^{-1}$ [1]	$k_1^{'} = \frac{k_4 T}{K_4}$	$k_4^{'} = 7810$ [1]	$k_4^{'} = 7.81 \cdot 10^4$ [1]
Ca^{2+} leak rate	$k_5 = 10^{-7}\,\mu M^{-1} s^{-1}$ [1]	$k_5^{'} = k_5 T K_4$	$k_5^{'} = 1.6 \cdot 10^{-5}$ [1]	
Ca^{2+} buffering rate	$k_6 = 100\,\mu M^{-1} s^{-1}$ [1]			
Ca^{2+} debuffering rate	$k_7 = 300\,s^{-1}$ [1]			
Ratio of Ca^{2+} buffering rates		$k_b^{'} = \frac{k_7 T}{k_6 K_4}$	$k_b = 9.38$ [1]	
Ca^{2+} efflux rate	$k_8 = 24.7\,\mu M s^{-1}$ [1]	$k_8^{'} = \frac{k_8 T}{K_4}$	$k_8^{'} = 38600$ [1]	
Max. WSS-induced Ca^{2+} influx rate	$q_{max} = 17.6\,\mu M s^{-1}$ [1]	$q_{max}^{'} = \frac{q_{max} T}{K_4}$	$q_{max}^{'} =$ 27500 [1]	
CCE rate	$k_{CCE} = 8 \cdot 10^{-7}\,\mu M^{-1} s^{-1}$ [1]	$k_{CCE}^{'} = k_{CCE} T$[illegible]	$k_{CCE}^{'} = 1.28 \cdot 1$[illegible] [1]	

Resting cytosolic Ca^{2+} concentration	$Ca_0 = 0.1\ \mu M$ [1]	$Ca_0^{'} = \frac{Ca_0}{K_4}$	$Ca_0^{'} = 0.313$ [1]	
Resting stored Ca^{2+} concentration	$Ca_{s,0} = 2828\ \mu M$ [1]	$Ca_{s,0}^{'} = \frac{Ca_{s,0}}{K_4}$	$Ca_{s,0}^{'} = 8840$ [1]	
External Ca^{2+} concentration	$Ca_{ex} = 1500\ \mu M$ [1]	$Ca_{ex}^{'} = \frac{Ca_{ex}}{K_4}$	$Ca_{ex}^{'} = 4690$ [1]	
Concentration of Ca^{2+} buffering sites	$B_T = 120\ \mu M$ [1]	$B_T^{'} = \frac{B_T}{K_4}$	$B_T^{'} = 375$ [1]	
Michaelis–Menten constants	$K_{CICR} = 0\ \mu M$ [1]	$K_{CICR}^{'} = \frac{K_{CICR}}{K_4}$	0 [1]	
Michaelis–Menten constants	$K_1 = 0\ \mu M$ [1]	$K_1^{'} = \frac{K_1}{K_4}$	$K_1^{'} = 0$ [1]	$K_1^{'} = 1$ [1]
Michaelis–Menten constants	$K_2 = 0.2\ \mu M$ [1]	$K_2^{'} = \frac{K_2}{K_4}$	$K_2^{'} = 0.625$ [1]	
Michaelis–Menten constants	$K_3 = 0.15\ \mu M$ [1]	$K_3^{'} = \frac{K_3}{K_4}$	$K_3^{'} = 0.469$ [1]	
Michaelis–	$K_4 = 0.32\ \mu M$ [1]			

Menten constants				
Michaelis–Menten constants	$K_c = 0.026\ \mu M$ [1]	$K_c^{'} = \frac{K_c}{\phi_0}$	$K_c^{'} = 0.26$ [1]	
Membrane shear modulus	$\delta = 10^{-5}\ kg \cdot s^{-2}$ [1]	$\delta^{'} = \frac{\delta}{\rho U_0^2 l}$	$\delta^{'} = 2.63$ for artery (Plank's model) [1] $\delta^{'} = 126$ for capillary	
Density of water	$\rho = 1000\ kgm^{-3}$ [1]			
Reference velocity	$U_0 = 1.04 \cdot 10^{-2}\ ms^{-1}$ [1] for artery (Plank's model) $U_0 = 0.2\ ms^{-1}$ typical for artery $U_0 = 1.5 \cdot 10^{-3}\ ms^{-1}$ typical for capillary			
Cell length in direction of flow	$l = 3.5 \cdot 10^{-5}\ m$ [1]			
Plasma membrane load fraction			$\varepsilon^{'} = 0.1$ [1]	
Plasma membrane			$f_e^{'} = 0.0134$ [1]	

energy gating fraction				
Ratio of cytosolic and ER volumes			$V_r^{'} = 3.5$ [1]	
Strain energy density constant		$W_0^{'} = \frac{\rho U_0^2 l}{8kT_e N}$	$W_0^{'} = 111$ [1] for artery $W_0^{'} = 2.3$ for capillary	
Temperature	$T_e = 310\ K$			
Ca^{2+} channel area density	$N_0 = 10^{12}\ m^{-2}$ [1]			
Boltzmann constant	$k = 1.3807 \cdot 10^{-23}\ kg \cdot m^2 s^{-2} K^{-1}$			
time	t, s	$t^{'} = \frac{t}{T}$		
Reference timescale	$T = 500\ s$			
Characteristic WSS for ATP production	$\tau_m = 1\ kg \cdot m^{-1} s^{-2}$ [1]		$\tau_m^{'} = 9.22$ [1]	
WSS	$\tau =$ 1 Pa for artery $\tau =$ 0.1 Pa [84] for capillary	$\tau^{'} = \frac{\tau}{\rho U_0^2}$ [1]	$\tau^{'} =$ 9.2 for artery $\tau^{'} =$ 44 for	

			capillary	

First, we reproduce the results of Plank's model for artery without magnetic field influence in non-oscillatory regime. Then we calculate the influence of oscillating magnetic shear stress. The set of dimensionless parameters for 'non-oscillatory regime', 'artery' is given in Table S2.

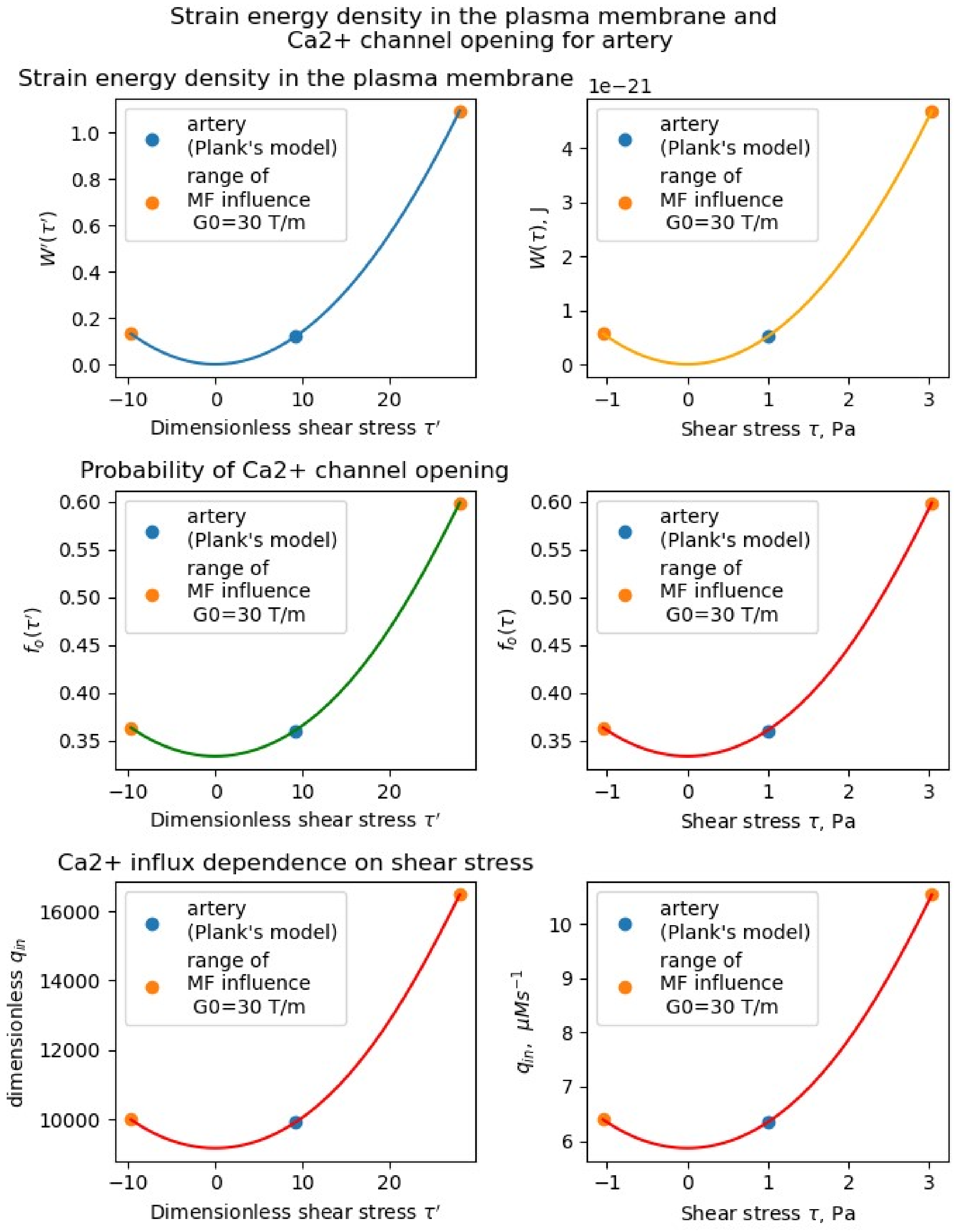
Strain energy density in the plasma membrane and
Ca2+ channel opening for artery
Strain energy density in the plasma membrane
Probability of Ca2+ channel opening
Ca2+ influx dependence on shear stress
artery
(Plank's model)
range of
MF influence
G0=30 T/m
1e−21
W′(τ′)
W(τ), J
f0(τ′)
f0(τ)
dimensionless qin
qin, μMs−1
Dimensionless shear stress τ′
Shear stress τ, Pa

Fig. S1 Range of oscillating magnetic field influence ($G_0 \in [0,30]\frac{T}{m}$) on strain energy density in the plasma membrane and Ca^{2+} channel opening for artery in Plank's model [1].

Code availability

The codes used in this study are available at www…. as .zip file containing the python project MagniCa.

Additional information

Supplementary information

The online version contains Supplementary Material available at www…..

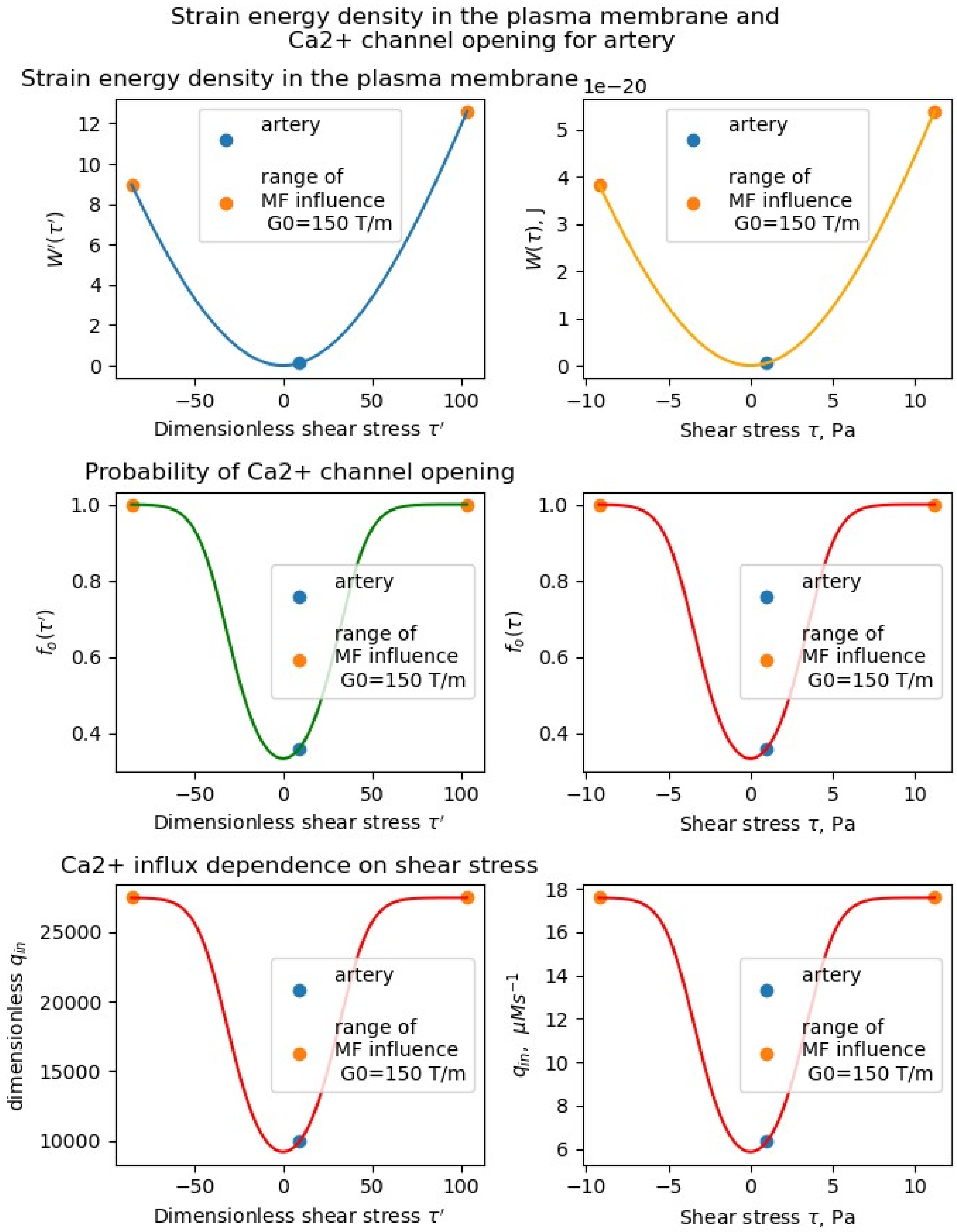
Strain energy density in the plasma membrane and
Ca2+ channel opening for artery
Strain energy density in the plasma membrane
1e−20
artery
range of
MF influence
G0=150 T/m
W′(τ′)
W(τ), J
Dimensionless shear stress τ′
Shear stress τ, Pa
Probability of Ca2+ channel opening
f0(τ′)
f0(τ)
Ca2+ influx dependence on shear stress
dimensionless q_in
q_in, μMs−1

Fig. S2. Range of oscillating magnetic field influence ($G_0 \in [0,150]\frac{T}{m}$) on strain energy density in the plasma membrane and Ca^{2+} channel opening for artery in Plank's model [1].

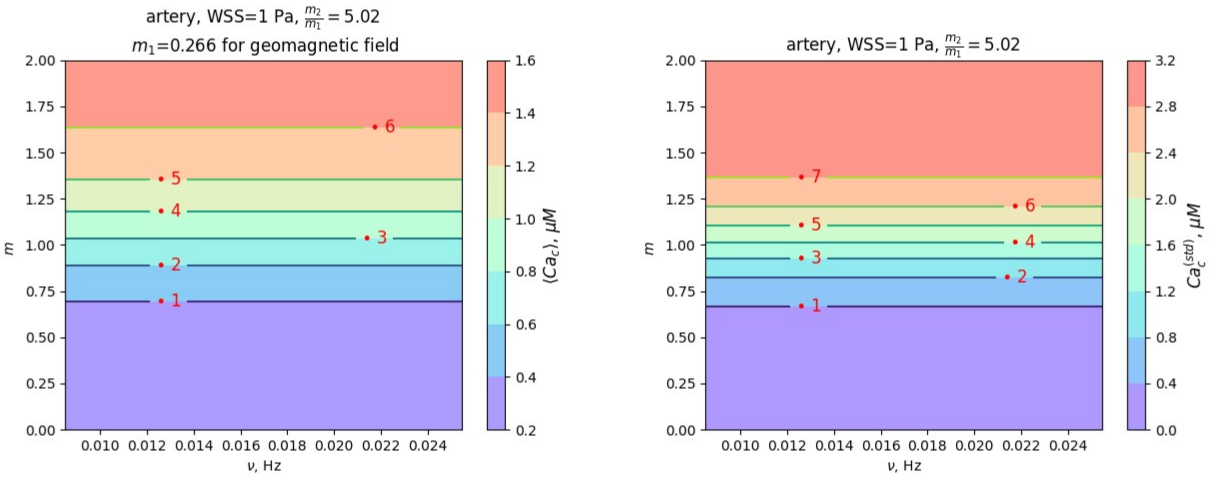

A

B

Fig. S3. Colormaps visualizing magnetic field effects on dynamics of intracellular free Ca^{2+} concentration for artery in non-oscillatory regime (Table S2).

WSSs created by magnetic field below "magnetic field saturation" threshold τ_{magn} ($G_0 < 80$ T/m in the case of gradient magnetic field) are much less than the normal blood pressure (1840 Pa in artery, upper limit of normal is 2660 Pa,[2] 4655 Pa in arteriolar;[3] capillary pressure differs markedly among tissues in the range of 665 Pa – 6650 Pa). Therefore, the direction of the magnetic force in plane of membrane is very important for ion channel gating. The out of plane magnetic force doesn't gate the ion channels as well as the out of plane force exerted on membrane by blood pressure itself doesn't gate the mechanosensitive ion channel.

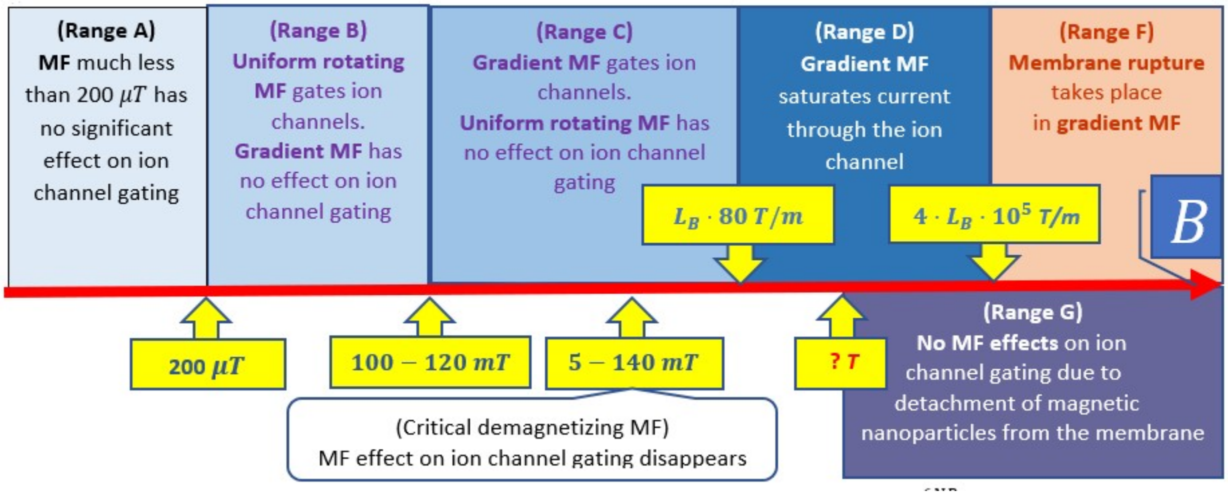

Fig. S4. **Roadmap of expected magnetic field effects. Range A:** Magnetic field (MF) $B \ll \frac{6NP_s}{\pi M_S}$ has no significant effect on ion channel gating because magnetic WSS is much less than WSS saturating Ca^{2+} ion channel. **Range B:** Uniform rotating MF gates ion channels. Gradient MF has no effect on ion channel gating for L_B greater than the length of chain of magnetic nanoparticles where L_B is the characteristic scale of magnetic field gradient. **Range C:** Gradient MF gates ion channels. Uniform rotating MF has no effect on ion channel gating because magnetic field is equal to saturation magnetic field for magnetic nanoparticle chain and the chain is magnetized parallel to the external magnetic field $B \approx 100 - 120\ mT$. **Range D:** WSS of gradient magnetic field $B_{cr} \approx L_B G_s$, $G_s \approx 80\ T/m$ saturates Ca^{2+} ion channel. **Range F:** Magnetic WSS is high resulting in membrane rupture $G_{cr} = \frac{3P_{cr}}{4rM_s} \approx 4 \cdot 10^5$ T/m, $B_{cr} = G_{cr} L_B$. **Range G:** No magnetic field effects on ion channel gating due to detachment of magnetic nanoparticles from the membrane.

References

1 Plank M.J., Wall D. J. N., and David T, Atherosclerosis and Calcium Signalling in Endothelial Cells, Prog. Biophys. Mol. Biol. 2006, **91**, 287.

2 L. Xue, Y. Yang, B. Sun, B. Liu, Q. Zeng and C. Xiong, Mildly Elevated Pulmonary Arterial Pressure Is Associated With a High Risk of Progression to Pulmonary Hypertension and Increased Mortality: A Systematic Review and Meta-Analysis, *J. Am. Heart Assoc.*, 2021, **10**, e018374.

3 M. J. Davis, M. A. Hill, and L. Kuo, Local Regulation of Microvascular Perfusion, in Microcirculation, Elsevier, 2008, , pp. 161–284.
